# Assessing the impact of a cleaning programme on environmental hygiene in labour and neonatal wards: an exploratory study in The Gambia

**DOI:** 10.1186/s13756-024-01393-6

**Published:** 2024-04-08

**Authors:** Uduak Okomo, Giorgia Gon, Saffiatou Darboe, Isatou C. M. Sey, Oluwatosin Nkereuwem, Lamin Leigh, Nfamara Camara, Lamin Makalo, Abdoulie Keita, Stephanie J. Dancer, Wendy Graham, Alexander M. Aiken

**Affiliations:** 1 Vaccines and Immunity Theme, MRC Unit The Gambia at LSHTM, Atlantic Boulevard, Fajara, The Gambia; 2 MARCH Centre, London School of Hygiene and Tropical Medicine, London, UK; 3 Infectious Disease Epidemiology Department, London School of Hygiene and Tropical Medicine, London, UK; 4 Research Microbiology Laboratory, MRC Unit The Gambia at LSHTM, Fajara, The Gambia; 5 AMR Centre, London School of Hygiene and Tropical Medicine, London, UK; 6 Department of Paediatrics, Edward Francis Small Teaching Hospital, Banjul, The Gambia; 7 Department of Obstetrics and Gynaecology, Edward Francis Small Teaching Hospital, Banjul, The Gambia; 8 Department of Microbiology, NHS Lanarkshire and School of Applied Sciences, Edinburgh Napier University, Edinburgh, UK

**Keywords:** Low-and-middle-income countries, Environmental hygiene, Cleaning, Neonate, Labour ward, Training, Intervention

## Abstract

**Background:**

Effective surface cleaning in hospitals is crucial to prevent the transmission of pathogens. However, hospitals in low- and middle-income countries face cleaning challenges due to limited resources and inadequate training.

**Methods:**

We assessed the effectiveness of a modified TEACH CLEAN programme for trainers in reducing surface microbiological contamination in the newborn unit of a tertiary referral hospital in The Gambia. We utilised a quasi-experimental design and compared data against those from the labour ward. Direct observations of cleaning practices and key informant interviews were also conducted to clarify the programme's impact.

**Results:**

Between July and September 2021 (pre-intervention) and October and December 2021 (post-intervention), weekly surface sampling was performed in the newborn unit and labour ward. The training package was delivered in October 2021, after which their surface microbiological contamination deteriorated in both clinical settings. While some cleaning standards improved, critical aspects such as using fresh cleaning cloths and the one-swipe method did not. Interviews with senior departmental and hospital management staff revealed ongoing challenges in the health system that hindered the ability to improve cleaning practices, including COVID-19, understaffing, disruptions to water supply and shortages of cleaning materials.

**Conclusions:**

Keeping a hospital clean is fundamental to good care, but training hospital cleaning staff in this low-income country neonatal unit failed to reduce surface contamination levels. Further qualitative investigation revealed multiple external factors that challenged any possible impact of the cleaning programme. Further work is needed to address barriers to hospital cleaning in low-income hospitals.

**Supplementary Information:**

The online version contains supplementary material available at 10.1186/s13756-024-01393-6.

## Introduction

Hospital-acquired infections (HAIs) pose a challenge, particularly for countries with limited healthcare resources for surveillance and infection prevention and control (IPC) [1]. The hospital environment acts as a reservoir for pathogens, which can persist for weeks or months on predominantly frequently touched near-patient surfaces posing a transmission risk for patients and staff [2–4]. Environmental hygiene is, therefore, integral to infection control and has been shown to impact rates of HAIs. Systematic removal of microorganisms from hospital surfaces through targeted and frequent cleaning reduces the bioburden and associated HAI risk [5–7]. However, cleaning practices in hospitals are highly variable, even within the same facility, and depend on staff availability, knowledge and skills, resources and managerial support. This variability creates confusion, neglect and missed opportunities for cleaning surfaces and equipment [8, 9]. Inadequate IPC training for non-clinical workers, including cleaning staff, further destroys incentives for good cleaning [10].

Babies born in hospitals in African settings, including those admitted to neonatal units, have a higher chance of developing HAIs due to poor practices and contaminated surfaces, overcrowded and understaffed wards, frequently shared or reused equipment and limited implementation of IPC measures [11–13]. Given the limited treatment options and increased morbidity and mortality associated with these infections, optimising IPC practices, including cleaning to reduce the bioburden of potential pathogens and HAI risk, is crucial.

TEACH CLEAN is a publicly available intervention package to improve environmental hygiene in low-resource maternity units by training cleaning staff, initially targeting maternity wards. However, cleaning in newborn care units has significant technical differences from cleaning in other hospital areas, as the intense vulnerability of newborns and the delicacy of medical equipment used during their care necessitate using different cleaning methods and chemical agents [14]. Thus, we adapted the TEACH CLEAN package for use in neonatal units in sub-Saharan Africa. In this paper, we report the results of a pilot study assessing the impact of the Neonatal TEACH CLEAN training package intervention on the adequacy of routine environmental cleaning in the neonatal unit of The Gambia’s largest hospital.

## Methods

### Neonatal teach clean training intervention

TEACH CLEAN is an education intervention aimed at improving environmental hygiene by training cleaning staff, with an initial focus on maternity units in low-resource settings. It was made publicly available in 2018 after piloting in The Gambia, India, and Cameroun and evaluation in Tanzania [7]. Key features of the training materials include participatory methods and pictorial guidelines to facilitate learning for cleaners with low education and literacy levels [15]. At the time of TEACH CLEAN's release, no training programmes were available for this cadre. This programme has since been adapted and used in many low-income countries and was recently adopted by the WHO as the basis for their own cleaners’ training resources [16, 17]. The TEACH CLEAN program consisted of seven modules covering crucial topics like personal hygiene, dress code, hand hygiene, PPE, housekeeping, waste handling, and linen handling. The program was designed to be taught by a designated trainer in the health facility, who was usually a healthcare professional in a leadership role in a clinical area. The trainer would receive training from a supervisor, also known as the 'master trainer,' from the district or regional level, using a "train the trainer" approach. In 2018, after the initial pilot of TEACH CLEAN, three additional modules were added to the original seven. These modules aimed to assist trainers and master trainers in learning to train, supervise, and establish quality improvement. In all applications of TEACH CLEAN and now the WHO resource, there is a crucial local adaptation phase when the generic guidance on frequency of cleaning and priority surfaces are contextualised to the specific healthcare setting, both to be consistent with existing IPC protocols in place and to allow for human resources availability. The adaptation is informed by the conduct of a local needs assessment exercise to identify existing practices, guidelines, previous training, numbers and literacy of cleaners, etc.

For the current study an additional training module was created for the original TEACH CLEAN package, focussing specifically on the peculiarities of surface cleaning in a neonatal unit. For ease of access, this is available in the Supplementary material for the paper. Healthcare professionals conducted local adaptation from a local NGO (Horizons Trust Gambia; HTG) that had previously worked with TEACH CLEAN. The participatory training included modules that covered not only cleaning practices for specific surfaces and preparation of cleaning fluids and materials but also priority surfaces for cleaning and frequency within a daily schedule. An additional module was prepared specifically for the neonatal unit, and it adapted the definition of the “patient zone” for the context of newborn cots. After the training, the cleaners were followed up with supportive supervision by the trainer and the senior staff in the neonatal unit. Pictorial guidelines on cleaning practices and frequency were also made available to them on the ward. These partners provided one master trainer to train four healthcare professionals from the neonatal unit to deliver the entire TEACH CLEAN package, plus the supplementary module for neonatal units. The training of cleaners from this clinical area was then conducted through workshops by the trainers at the participating hospital in October 2021. The master trainer from HTG also attended and provided further support to the trainers, particularly around supportive supervision, during the month after the cleaners’ workshop. According to the study design, the control (labour ward) did not receive training during the intervention. A similar training-the-training approach was delivered by HTG for the control ward after the data collection was completed. The same microbiological samples and qualitative data capture were conducted in the intervention (neonatal unit) and control (labour ward) areas.

### Study design, setting and population

We conducted a mixed-methods study at the neonatal unit of a large government tertiary referral hospital in The Gambia, the Edward Francis Small Teaching Hospital (EFSTH). This work was conducted as a collaboration between the MRC Unit The Gambia at the London School of Hygiene & Tropical Medicine (MRCG at LSHTM), LSHTM and the Epidemiology and Disease Control Unit (IPC Unit) of the Gambian Ministry of Health. The EFSTH is the sole national teaching and tertiary government referral hospital of the Gambia. With approximately 3,000 deliveries per year, the maternity unit at EFSTH has the third-highest annual deliveries nationwide. The neonatal unit at EFSTH has 30 cots, including an acute care ward, two low-risk wards, and a Kangaroo Mother Care (KMC) ward with six beds. During peak admission periods, cot occupancy is often over 200%, since neonates share cots and incubators [18]. This pilot study focused on neonatal acute care, KMC wards, and the labour ward as a comparator area. Our quasi-experimental study design aimed to evaluate the impact of the training intervention on cleaning behaviour and techniques and surface microbiological cleanliness. We also collected qualitative data on health system barriers to ensuring environmental hygiene in the facility and the training experience.

### Methods used to assess the adequacy of routine environmental cleaning

Throughout our study, we monitored the cleanliness of hospital ward surfaces weekly. To accurately measure aerobic colony counts (ACC) and identify Staphylococcus aureus, a key indicator pathogen for neonatal sepsis in sub-Saharan Africa [19], we utilized double-sided dipslides coated with nutrient and staphylococcal selective (Baird-Parker) agars. This was carried out during the baseline period (July to mid-September 2021) and post-training intervention period (mid-October to December 2021) in both intervention and control wards. Before collecting dipslides, we carefully observed hospital staff to determine which surfaces were the most frequently touched by healthcare workers. We then selected sampling locations in advance and created a list of key high-touch surfaces. Samples were taken from the same locations weekly but on a randomly selected day each week. (Supplementary Table A1). We tried to collect samples from equivalent positions of objects, especially cots and beds, every week, although there was some equipment movement around the ward between weeks. The same staff members took the samples weekly and were instructed to approach the sample collection consistently throughout the study. We ensured we did not leave any permanent marks on the objects while collecting samples. We followed the manufacturer's instructions for sample collection, after which dipslides were transported to the MRCG-at-LSHTM microbiology laboratory for processing. They were incubated aerobically at 40ºC for 48 h. Trained staff read dipslides and quantified aerobic growth as follows: 0 CFU/cm^2^; 0–2.5 CFU/cm^2^; 2.5–12 CFU/cm^2^; 12–40 CFU/cm^2^; ≥ 40 CFU/cm^2^; and confluent growth. To ensure accuracy, 10% of the dipslides were read by a second reader and 5% by a third reader, with any discrepancies re-evaluated. We searched for the presence of *S. aureus* based on colonial appearance and subculture on blood agar with a positive coagulase test (Pro-Lab Diagnostics, UK). Confirmatory testing for possible *S. aureus* was performed with a positive DNAse test. We classified this organism as either "present" or "absent" on each dipslide.

#### Observation of cleaning practice

A single data collector observed cleaning episodes once a week for 14 weeks in the neonatal unit (intervention) and labour ward (control) after the intervention. These observations were conducted early on Wednesday mornings to ensure consistency. We attempted to conceal the study's true purpose from staff by framing it as an assessment of overall care quality. We recorded various aspects of cleaning techniques, such as whether the cleaning staff covered all regions of the patient zone during the cleaning process, used a fresh cloth, and allowed the area to dry before use (Supplementary Table A2). The observation was explicitly concentrated on cleaning the patient zone, including the bed and other items designated for that area. It focussed on the same surfaces and practices each week.

#### Qualitative interviews and group discussions

We conducted key informant interviews with several individuals to gain insight into the factors affecting the cleaning of hospital wards. These included the chief matron, the matron responsible for the neonatal unit, the head of domestic services (also known as cleaners or orderlies), and the head of the hospital's IPC Unit.

### Statistical methods

Based on previous work conducted in maternity wards in Tanzania, we anticipated that the training intervention would enhance the dipslide ACC "pass" rate from approximately 25% to approximately 50% [7]. The sample size used in this study had more than 99% power to detect a change in that scale. It was intentionally overpowered to detect changes in underlying trends, including the Hawthorne effect. The data were cleaned to ensure accuracy, consistency, and completeness before being analysed using Stata software. The degree of surface cleanliness was categorised into five groups following the laboratory classifications. Based on previous research, we also categorised the ACC results into "pass" for those below 2.5 CFU/cm^2^ and "fail" for those at ≥ 2.5 CFU/cm.^2^ [20]. Key informant interviews were recorded in English and one of the local languages (Mandinka) using a digital recorder. We used NVivo 12 software to manage and code transcripts of the audio recordings. The thematic analysis process involved several steps: familiarisation with the data by relistening to the audio recordings and/or re-reading the transcripts and observation field notes; initial coding and searching for themes; and review, definition, and naming of themes [21].

## Results

**Fig. 1:**
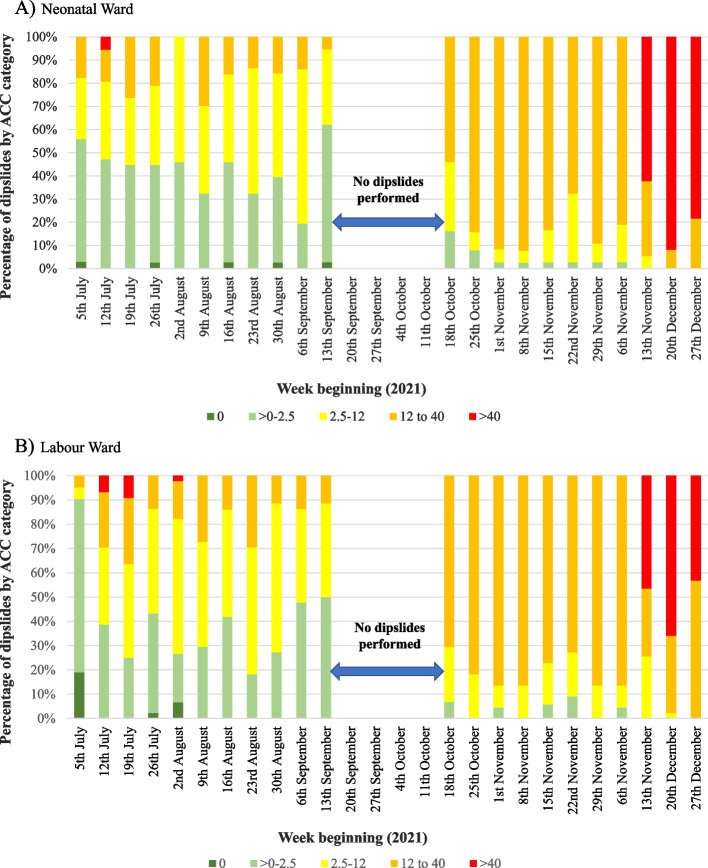
Dipslide aerobic colony count (ACC) categories in the neonatal and labour wards

The study was conducted between July and December 2021, with data collected before and after the intervention. The baseline data collection period lasted 10 weeks, from July 5th to September 20th, while the post-intervention period lasted 7 weeks, from October 18th to December 27th. In the neonatal ward, 813 dipslides were used to measure surface contamination. We also collected 956 dipslides from the labour ward. The median number (range) of dipslides collected from each ward during weekly data collection was 37 dipslides (34–39) in the neonatal ward and 44 dipslides (35–45) in the labour ward. Figure 1 shows the weekly categories of ACC results for the neonatal and labour wards. During the baseline period between July and September 2021, there was a gradual decline in the proportion of dipslides with less contamination (0 and 0–2.5 CFU/cm^2^) in both wards. In October and November, during the post-intervention period, there was an increase in the proportions of dipslides exhibiting higher degrees of contamination. In both wards in December, there was a significant increase in the proportion of dipslides exceeding 40 CFU/cm^2^.

The proportion of dipslides achieving a pass (ACC < 2.5 CFU; absence of *S. aureus*) in the neonatal ward during the baseline and post-intervention periods are shown in Table 1.

**Table 1:** Dipslide results from EFSTH Neonatal Unit and Labour ward

	**Neonatal unit** **(intervention)**		**Labour ward** **(control)**	
	**“Pass” n/N**	**Percentage pass**	**“Pass” n/N**	**Percentage pass**
**Aerobic Colony Count (ACC), “pass” is ≤ 2.5 CFU/cm**^**2**^				
Baseline period (July–September 2021)	173/405	43%	191/482	40%
Post-intervention period (Oct- December 2021)	15/408	4%	13/474	3%
** Change**		**-39%**		**-37%**
**S. aureus detection, “pass” is absence of organism**				
Baseline period (July–September 2021)	359/405	89%	448/482	93%
Post-intervention period (Oct- December 2021)	358/408	88%	429/474	91%
** Change**		**-1%**		**-2%**

For the ACC evaluation, there was a dramatic decline in the proportion of dipslides samples that achieved a pass level in both wards, whereas, for *S. aureus* evaluation, the results were similar between wards and study periods. The decline in the ACC pass rate represents a deterioration in microbiological cleanliness from the baseline to post-intervention periods. This dramatic worsening of microbiological cleanliness was seen in both the intervention (neonatal unit) and control (labour ward) areas.

### Cleaning practices

We observed 65 cleaning procedures in the neonatal ward and 50 cleaning procedures in the labour wards. Most (93%) of the cleaning procedures were done by cleaners and nurses. Improvement was noted in four categories out of the 13 assessed (Table 2). However, other crucial aspects of the technique, such as using fresh cleaning cloths and the one-swipe method, did not improve.

**Table 2:** Cleaning procedure categories with observed improvement

Variables	Neonatal (*N* = 65) N (%)	Labour (*N* = 50) N (%)	Chi square *p*-value
PPE worn	64 (99)	46 (92)	0.092
Patient presence	2 (3)	12 (24)	0.001
Cleaned all surfaces	64 (99)	46 (92)	0.099
Left area to dry	58 (89)	37 (78)	0.078

#### Qualitative interviews and group discussions

During interviews with senior nursing, domestic services, and hospital IPC unit staff members, it was acknowledged that maintaining cleanliness in the hospital is important and requires shared responsibility. It was agreed that the primary responsibility for ensuring the cleanliness of the hospital environment falls on the domestic service staff, namely orderlies or cleaners. A male staff member emphasized the significance of their role in supporting the quality of service provided by doctors and nurses in the hospital wards:

*“We are committed to keeping the premises clean. A clean environment is essential for doctors and nurses to work effectively, so we ensure the entire area is thoroughly cleaned before they begin their morning workday.*” ***[Senior domestic services staff member]***

He also stressed the significance of keeping the hospital environment clean to reduce the spread of infections,

*“… It is important to maintain a clean environment to eliminate bacteria and provide a healthy space for admitted patients.” ****[Senior domestic services staff member]***

During interviews with the department's senior administrative staff and hospital nursing staff, it was disclosed that the Department of Paediatrics had been experiencing a chronic shortage of nursing and cleaning staff.

“There has been a long-standing shortage of nursing staff in the neonatal unit. Ideally, the nurse-to-newborn ratio should be 1:1 or 1:2. Unfortunately, during each shift, only a maximum of 6 trained personnel is available to care for 48 or more babies.” ***[Senior hospital nursing administrative staff member]***

During the study period, several factors worsened the staff shortage. Some staff members were on maternity leave, while others could not work due to COVID-19. Some employees had retired, leaving vacant positions. Furthermore, the Department of Paediatrics required more staff due to ongoing renovations and expansion work.

*“At times, two cleaners may be present in the neonatal unit during the morning shift, but only one cleaner during the afternoon and night shifts. This sole cleaner is responsible for cleaning the neonatal unit and completing other tasks, such as obtaining medications from the hospital pharmacy and collecting supplies from various departments. Unfortunately, there was a period during the COVID pandemic when one of the cleaners tested positive for the virus. ****[Senior departmental nursing administrative staff member]***

It was observed that there was a severe lack of nurses and cleaners in hospital wards, which negatively affected the quality-of-service delivery. This resulted in numerous unpleasant experiences for both healthcare workers and patients. The delay in addressing staffing issues increased the workload and inefficiency of the staff in the hospital wards.

“Due to the shortage of nursing staff, the quality of work was compromised, and the level of care provided was not up to expectations. The few staff members available were overworked and burnt out.”*** [Senior hospital nursing administrative staff member]***

It was just the two of us; honestly, we couldn’t keep the whole place clean. It was very challenging for us to keep everything tidy, and even the nurses struggled to cope. Unfortunately, we experienced many deaths during that time, which I think was because we did not have enough staff.” ***[Senior domestic services staff member]***

During the discussion, some staff brought up the ongoing problems with water supply throughout the hospital and a shortage of cleaning supplies. One participant specifically mentioned these issues:

*"We frequently experience problems with water supply at the hospital, as it can be inconsistent."* ***[Senior nursing administrative staff member]***

One of the staff interviewed thoroughly explained the difficulties they faced in obtaining necessary cleaning supplies:

“We ran out of supplies, particularly bleach, which was not good because you cannot clean without bleach for infection control purposes. We usually use dilute bleach as a part of our daily cleaning to disinfect the cots before each admission and after each discharge. We know the percentage dilution and dilute it ourselves. During the shortages, I had to seek out donations to provide us with something to use before the hospital supplies became available. Even when the hospital restocks, the quantity is limited, and we must prioritise cleaning the toilets first before the ward”. ***[Departmental nursing administrative staff member]***

Certain staff members remembered difficulties keeping the hospital environment clean during the study period. This was particularly challenging when several areas in the hospital, including the neonatal ward and Department of Paediatrics, underwent renovations, causing significant disruptions to the usual operations:

*“During the renovation work in the neonatal unit, cleaning was disrupted and became sub-optimal and inefficient due to dust. As a result, many areas were left uncleaned during those days. Additionally, the unit became congested due to the movement of items required for the paintwork.”.* ***[nursing administrative staff member]***

The staff highlighted the significance of training and retraining the cleaning staff, stressing its positive impact on the quality of services provided. Specifically, the previous training received by the cleaning staff on the maternity ward greatly improved their performance:

*“We underwent training on maintaining cleanliness and tidiness in hospital facilities while assisting babies and patients.”* ***[Senior domestic services staff member]***

## Discussion

We undertook a quasi-experimental intervention study to measure changes in surface microbiological contamination in the neonatal unit and labour ward of the national teaching and referral hospital in Gambia, a low-income country. As an intervention, we adapted a training package for hospital environmental cleaning for use in a neonatal unit. We worked with local partners to optimise the delivery of these participatory training materials using a training-of-trainers approach. Contrary to our expectations, environmental contamination in the neonatal unit, objectively measured as the total ACC on dipslides, worsened rather than improved after the intervention. There was no change in the prevalence of *S.aureus* on surfaces and only relatively modest changes in actual cleaning techniques from watching cleaners.

Cleaning involves a series of procedures and practices rather than a single technique. It is important to note that while some procedures may have improved after training, others—particularly the most important ones—may not have improved, which could have affected the effectiveness of the intervention in this study. Despite some improvements, the intervention wards did not differ significantly from the control wards. Our study reveals that the health system challenges ongoing during the study period may have outweighed any benefits of the intervention.

We examined different possible mechanisms for the decline in hospital surface cleanliness. One possibility we considered was that the more intense mechanical cleaning led to greater biofilm disruption. Biofilm bacteria are more tolerant to cleaning agents and disinfectants, especially chlorine disinfectants [22, 23]. However, using aggressive cleaning methods or disinfectants can harm the microbial community and the structure supporting the biofilm, releasing a larger number of viable microorganisms and increasing the detection of multidrug-resistant organisms [24, 25]. However, this explanation was unlikely, especially since the contamination continued to worsen even after the cleaning intervention had been in place for some time.

Maintaining a clean environment varies from the principles of clinical care interventions. The differences between cleaning and patient care have a behavioural element that is magnified within the context of patient care. Clinical staff receive training to recognize the need for hygiene when they encounter a patient or perform a clinical intervention. On the other hand, cleaners may not have received the same level of training as hygiene is a critical element that is not always emphasized in cleaning tasks. Therefore, a cleaner wiping down the top of a patient's locker, table, or bed frame may not have had detailed training on cleaning a high-risk surface or even recognizing the direct importance of their actions to the patient's infection risk. This may result in priority cleaning areas near patients being overlooked. Cleaning staff should understand the context and purpose of each cleaning task. By conducting a thorough risk assessment of surfaces that require cleaning and identifying the risk associated with each surface, efforts can be focussed on areas most likely to pose a high risk of infection to patients. This targeted approach can effectively help to prevent the spread of infection [26]. During our study, we selected and examined all the high-touch areas near the patients considered high-risk for exposure. These areas were given priority for cleaning. The cleaning staff who were the focus of the intervention may have had some difficulty distinguishing between general cleaning and hygiene protocols. This may have contributed to the less-than-optimal outcomes observed following the TEACH CLEAN training intervention. Nevertheless, we remain confident in the program's effectiveness, as it was specifically designed with low-literacy cleaners in mind and any potential risks were deemed negligible.

We looked for any potential time-related factors that could have led to increased contamination measurements after the intervention. It is improbable that modifications in laboratory measurements of the microbiological outcome over time caused this effect. Dipslide samples were gathered and handled with a standardised procedure by the same team of experienced staff in a quality-controlled lab throughout the study. We also considered the potential contribution of seasonal climatic variations to the observed effect, as studies of microbial contamination of rivers and surface water have typically shown that periods of higher rainfall are associated with higher water contamination levels after a short lag [27]. The Gambia experiences two distinct seasons throughout the year. These include a long dry season from November to May and a short wet season from June to October. During the dry season, temperatures range from 18 °C to 30 °C, while during the wet season, temperatures range from 23 °C to 33 °C. We analysed publicly available temperature and rainfall data over the study period for the City of Banjul. The study's post-intervention period overlapped with the Gambia's cooler, dry season. We judged that lower humidity and temperatures would be less conducive to bacterial growth on surfaces hence, the dry season appeared unlikely to be responsible for the observed effects. Additionally, we considered the possibility of limitations on municipal water supply in Banjul during the drier period of the year, leading to cleaners being exposed to a more contaminated water supply. This seemed more plausible, but we could not confirm this hypothesis without retrospective hospital water quality data.

Finally, we considered the potential for cross-contamination from using the same cleaning equipment and cloth to clean multiple areas. Adequate cleaning and disinfection protocols have been identified as effective measures to minimize the risk of cross-contamination. Despite implementing a training program to enhance our staff's cleaning practices, we recognize that limited resources may have impacted the sustainability of the intervention, especially as our intervention did not provide additional cleaning resources.. According to the COM-B model [28], behavioural change is influenced by various factors, with capability (C), opportunity (O), and motivation (M) being the most significant. Capability refers to an individual's mental and physical ability to engage in an activity. Opportunity refers to external factors that enable a behaviour. Finally, motivation refers to conscious and unconscious cognitive processes that guide and inspire behaviour. At least one of these factors must be modified to change behaviour. Although the TEACH CLEAN training improved the staff's cleaning abilities in the neonatal unit, the opportunity or motivation to clean may not have improved. The key informant interviews provided insightful qualitative data on the staff's perspectives and experiences regarding obstacles and facilitators to maintaining hospital environmental hygiene and cleaning the wards. The health system barriers, particularly chronic understaffing of nurses and cleaners worsened by COVID-19, recurrent water supply disruptions, shortages of cleaning materials and consumables, and other practical obstacles to cleaning, were persistent and significant challenges that the training intervention could not overcome. This is consistent with similar challenges in the control ward, which faced the same staffing and infrastructural issues and experienced a similar decline in microbiological cleanliness.

Our study has some limitations. Firstly, we could not observe the cleaning practices before implementing our intervention in the neonatal ward. Secondly, the time we had to collect dipslides after the intervention was shorter than the time we spent observing the cleaning procedures. Therefore, we may not have been able to determine the intervention's effect on surface microbiological contamination accurately. Additionally, due to the need for more intensive observation work, we required additional resources to gather detailed data on cleaning practices, including the frequency and quality of cleaning fluid preparation. Qualitative interviews were conducted with four senior individuals instead of the cleaning staff on the ward. These interviews were part of a formal research study conducted in a hospital known for having issues with the transmission of infections in the neonatal unit. While there may have been some social desirability bias during the interviews, with staff members potentially providing answers they believed the researchers wanted to hear, informal conversations with cleaners suggest that some of them may have been aware of the true nature of the observations.

## Conclusions

The importance of maintaining a clean environment in hospitals, particularly in areas where high-risk patients are present, cannot be overstated. This is especially crucial in low-income hospital settings. Our study centred around a training program for hospital cleaners in a hospital located in the Gambia. While surface microbiological contamination did not show objective improvements, this study represents one of the pioneering efforts to evaluate the cleaning quality of a neonatal unit in a low-resource setting. We strongly recommend ongoing training and support for low-income hospital cleaners, coupled with objective assessments to determine the impact of such training on overall cleanliness. However, investing limited resources in reducing infections through environmental cleanliness may not yield the desired results without adequate cleaning supplies and resources. One potential solution is to explore how cleaning efforts can be optimized for better focus and effectiveness. This targeted approach can effectively help to prevent the spread of infection. Additional research in this area could uncover valuable insights into the potential benefits of such an approach and help guide policy decisions regarding resource allocation in the fight against infection.

### Supplementary Information

**Supplementary Material 1.**

**Supplementary Material 2.**

## Data Availability

All data generated or analysed during this study are included in this published article [and its supplementary information files].

## Abbreviations

ACC: Aerobic colony count
CFU: Colony-forming Units
EFSTH: Edward Francis Small Hospital
HAI: Hospital-acquired infections
IPC: Infection Prevention and Control
KMC: Kangaroo Mother Care
