# Supplementary material for: Assessing the impact of a cleaning programme on environmental hygiene in labour and neonatal wards: an exploratory study in The Gambia
Source: Antimicrob Resist Infect Control. 2024 Apr 8;13:36. doi: 10.1186/s13756-024-01393-6 (PMC11003010; doi:10.1186/s13756-024-01393-6)
Supplement: Supplementary file 1 — Supplementary Material 1. [file 13756_2024_1393_MOESM1_ESM.pdf]

TEACHCLEAN: Training in Environmental Hygiene and Cleaning in Healthcare

Supplementary Module C (CLEAN BOX): Cleaning the neonatal unit

October 2021

Learning objectives - on completion of this module, participants should be able to:

- Understand the importance of maintaining standards of environmental hygiene in the neonatal unit
- Identify procedures and practises crucial to these standards
- Recognise the need for parents to play their part in keeping the neonatal unit safe

The module comprises five main sections:

1. Specific hygiene challenges of neonatal units
2. The "patient zone" in neonatal units
3. Different roles of different staff in maintaining environmental hygiene in neonatal units
4. Schedule for cleaning
5. Involvement of parents

Contents of the Clean Box:

- A copy of these instructions
- Two photos of neonatal units for Discussion exercise I
- Picture of patient zone diagram to show participants from section 2
- Flip chart & pens to note problems and solution to Discussion exercise II
- Sufficient photos for each small group to have a set for Practical exercise I
- Print out copies of the Parents' Information sheet for Discussion exercise III

1. Specific hygiene challenges of neonatal units

Neonatal units have a particularly vulnerable patient group – newborns. In some facilities, these units are further divided into intensive care rooms for very sick babies (NICU), rooms for recovering babies, and spaces for mothers to nurse small babies using Kangaroo Mother Care. All these groups of newborns are at risk from healthcare-associated infections acquired from the physical environment (such as contaminated cots), from the medical procedures used to care for them (such as IV lines) and indeed from other babies (such as those entering the unit already with newborn sepsis), as well as from healthcare workers and visitors (such as via their contaminated hands). Infection prevention and control is thus a crucial activity in neonatal units and requires the adherence to clear guidelines or standard operating procedures. Neonatal intensive care units are similar to ICUs for other patient groups in needing particular attention to terminal cleaning procedures which must be applied after a patient leaves and before the bed/cot is re-occupied.

Many of the other key infection and prevention practises in neonatal units are common to all clinical areas, and users of this module should already have received learning from the other modules/clean boxes and from the Cleaning Procedures chapter of TEACH CLEAN in terms of:

- Personal hygiene & dress code
- Hand hygiene
- Personal protective equipment
- Housekeeping/control of environment
- Waste handling
- Linen handling

Discussion I

Ask participants to look at this photo of a neonatal unit in Mexico and discuss what makes cleaning particularly difficult here?

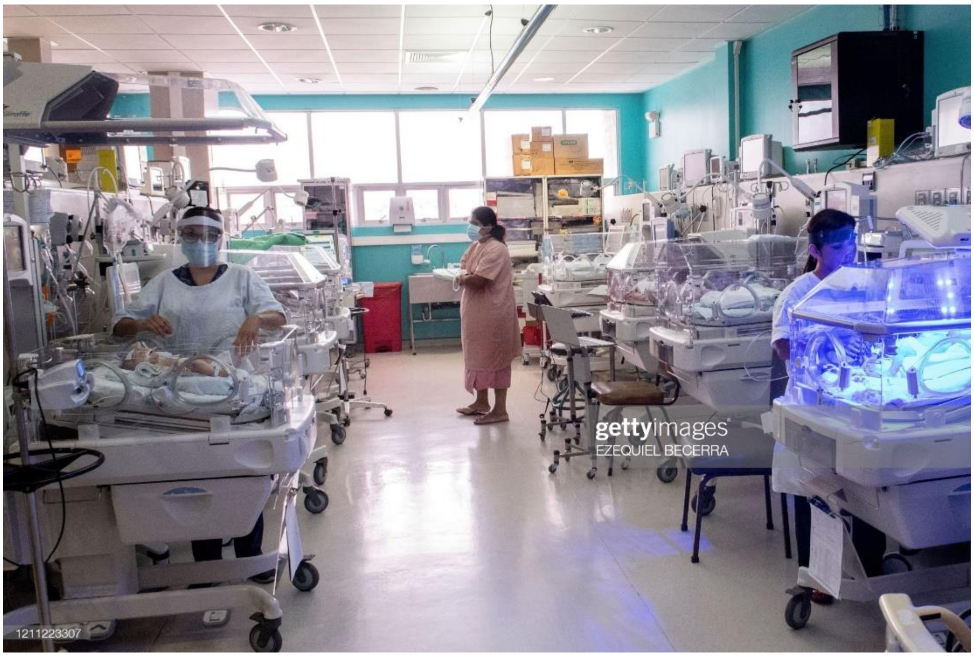

Discussion points:

- *Staff & mother*
- *Lots of medical equipment*
- *Limited space between cots*

Ask participants to then look at this photo from a neonatal unit in West Africa, and identify any further challenges.

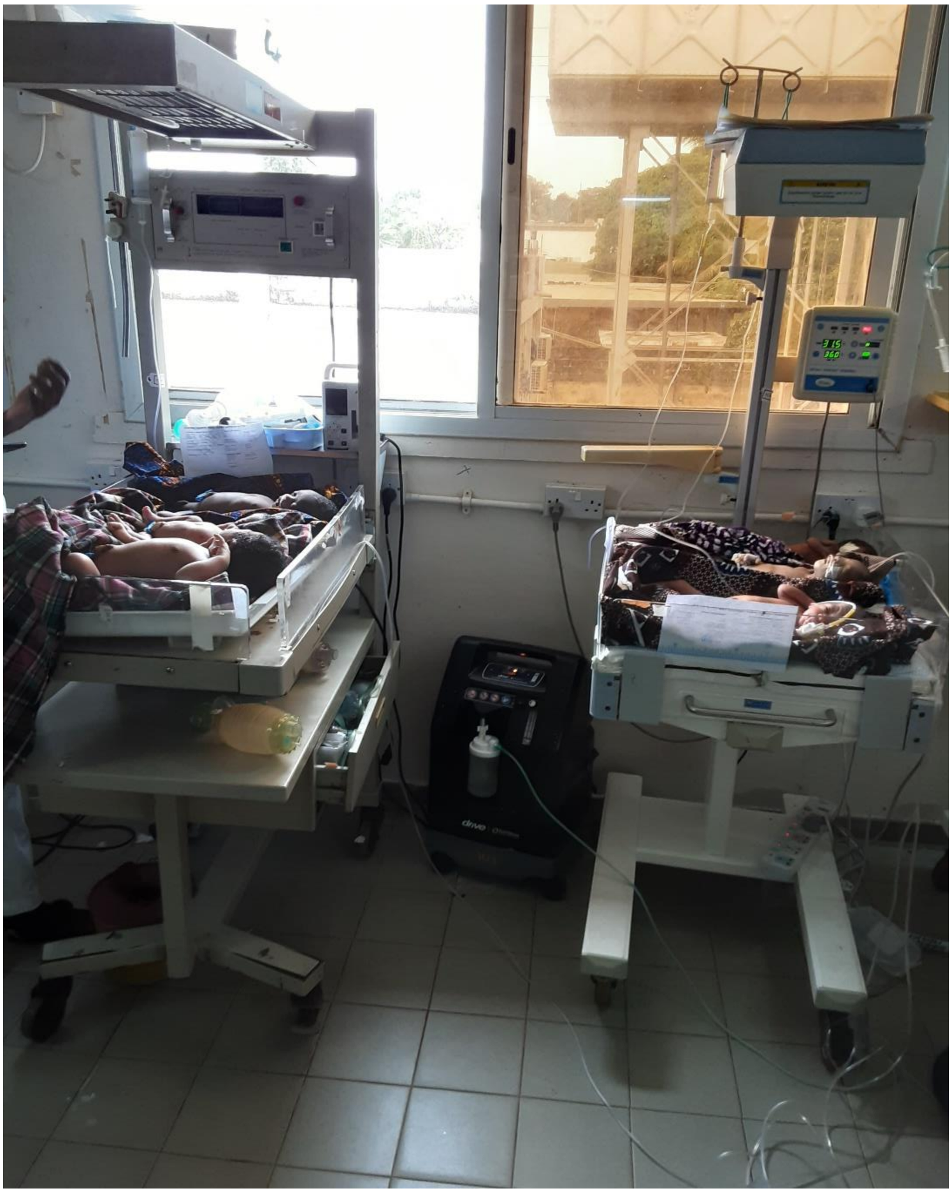

Other discussion points:

- *More than one baby per cot*
- *Tubing on the floor/floor area is congested*
- *Each baby has different linen (from home/not well-laundered?)*
- *Area is dark so hard to see for cleaning*

Besides the vulnerability of the patients in neonatal units, there are other factors peculiar to these clinical areas which affect cleaning frequency and methods:

- High turn-over of patients (newborns)
- High levels of equipment used to monitor and stabilize babies
- Prolonged presence in unit of parents as well as health workers
- High birth seasons mean overcrowding in neonatal units is acute at some times of year
- Continuous observation of babies (24/7)
- Newborns may undergo surgery as well as medical treatment, and face additional risks of post-surgical infections

2. What is the patient zone in neonatal units and why does it matter?

Definition:

*According to the World Health Organization (WHO), **the patient zone** contains the patient and his/her immediate. surroundings. This typically includes the intact skin of the patient and all inanimate surfaces that are touched by or in direct physical contact with the patient, such as the bed rails, bedside table, bed linen and infusion.*

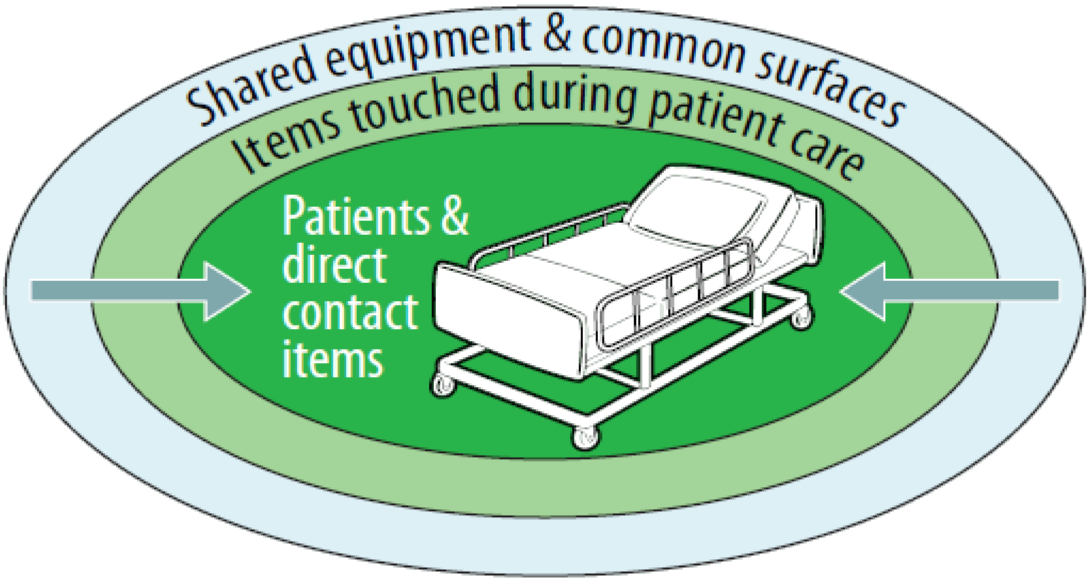

https://www.cdc.gov/hai/prevent/resource-limited/cleaning-procedures.html

This diagram shows the patient zone as the area in dark green in the centre – shown by the arrows. The two outer circles are referred to as the *healthcare zone*.

One of the most important reasons for defining the patient zone relates to hand hygiene practises, as discussed in an earlier module (Clean Box 3). The key times for hand hygiene are:

- on entering the patient zone (this includes after closing the screens/curtains as they are potentially contaminated)
- between touching the patient, touching the critical sites on the same patient (e.g. IV site and drain sites) and conducting an aseptic procedure
- before leaving the patient zone

? Cleaning the neonatal unit case study I

Adama had been sent to help clean the NICU, although she usually works on the maternity unit. She was used to going into the patient zone around the beds occupied by new mothers, and wiping the bed rails and washing the floor. Adama knew she should wash her hands before and after entering the patient zones, and to wear disposable gloves as she was handling a chlorine-based cleaning fluid. In the neonatal unit, things looked very different. There were many cots close together and several

babies in each cot. Adama was confused about what was the patient zone here. Can you help her by defining the zone and when she should practice hand hygiene?

Response: The patient zone is the same for neonatal units as for adult wards, but the difference can be that patients (babies) share the same zone. This means good hand hygiene is even more important as you can increase the infection risks for several babies at the same time through poor practise. Adama should practise hand hygiene before and after entering the patient zone.

3. Different roles of different staff in maintaining environmental hygiene in neonatal units

Just as in other clinical wards, maintaining good environmental hygiene in the neonatal units typically involves two main types of workers: healthcare professionals – such as nurses and midwives, and support staff – such as auxiliaries and cleaners. Although there may be some cleaning activities which overlap between these two, for example, wiping-down a bed or cot mattress, other activities are very clearly the role of one group, such as nurses cleaning medical equipment. It is really important that there are clear cleaning schedules (as discussed in the next section) and clear understanding of who is responsible for doing the cleaning and supervising the cleaning. This set-up helps to ensure high standards of hygiene are consistently maintained across the ward. When there are shortages of staff, it sometimes happens that tasks have to be shared differently, but it is always important to ensure that any tasks which are new to the staff are fully explained and supportively supervised.

Discussion II

Read out these cleaning activities, and ask participants who is usually responsible in their unit and who supervisors such tasks?

Cleaning Task	Personnel responsible for task	Personnel responsible for supervision of task
a) Cleaning of the inside of neonatal cots, including mattresses		
b) Cleaning of the outside of neonatal cots		
c) Cleaning of patient zone around the cots, incubators and resuscitaires, including chairs for mothers		
d) Changing of cot sheets		
e) Cleaning of the inside of incubators, including mattresses		
f) Cleaning of the outside of incubators		
g) Cleaning of the inside of resuscitaires, including mattresses		
h) Changing of the outside of resusitaire		

i) Floor cleaning		
j) Cleaning of nearest toilets/latrines for mothers		
k) Cleaning of nearest toilets/latrines for staff		
l) Cleaning of neonatal unit hand washing facilities for mothers		
m) Cleaning of neonatal unit hand washing facilities for staff		
n) Removal of clinical waste from the neonatal unit		
o) Removal of non-clinical waste from the neonatal unit		
p) Removal of sharps waste from the neonatal unit		
q) Cleaning of medical devices		

Discussion points

Try to make sure all participants take part, and avoid one person dominating the responses. Where there is uncertainty or disagreement, try to find out why this is the case and who should help to resolve it so that task roles are clear. Encourage participants both to share any problems they face in doing these tasks or in supervising them, and to identify possible solutions. This exercise is about creating a sense of team and collective effort in keeping the neonatal unit clean and safe.

4. Schedule for cleaning

Given the vulnerability to infection of the patient group in the neonatal unit and the other factors mentioned earlier – such as …. {*Trainer: encourage participants to call out*: e.g. high turn-over of newborns; high levels of equipment used to monitor and stabilize babies, etc – see page 2} - the frequency of cleaning is crucial to maintaining good standards of hygiene.

Generally speaking, there are four types of frequency of cleaning:

- After every patient contact
- Twice daily routinely
- After each patient has been discharged
- Weekly routinely

Practical I

Divide the participants into small groups (ideally less than 4 per group), and give each group a set of the 12 photos of equipment, surfaces, etc for this exercise.

Ask the group to place the photos in one of 4 piles to indicate the frequency.

After they have done this, ask each group in turn to call out where they have put a photo in terms of cleaning frequency, and check with other groups if they have done the same. If there is disagreement, discuss why there was a difference of opinion.

Make sure all participants are aware of the correct answers below:

Photo number	Image	Correct cleaning frequency				What to clean with
		After every patient contact/after every use	Twice daily	After patient discharge	Weekly	
1.	Stethoscope	X				Use 70% isopropyl alcohol solution on a cloth or pre-packaged wipe
2.	Floor		X			Detergent & water
3.	Cot outside		X			Chlorine-based disinfectant
4.	Thermometer	X				70% isopropyl alcohol solution on a cloth or pre-packaged wipe
5.	Walls				X	Detergent & water
6.	Staff hand-washing sink		X			Chlorine-based disinfectant
7.	Incubator inside			X		70% isopropyl alcohol solution on a cloth or pre-packaged wipe
8.	Window frames				X	Detergent & water
9.	Cleaning bucket	X				Detergent & water
10.	Weighing scales	X				70% isopropyl alcohol solution on a cloth or pre-packaged wipe
11.	Underside of cot frame			X		Chlorine-based disinfectant
12.	Chair for staff or visiting parents		X			Detergent & water

After they have completed the photo exercise, ask them to call out what they should use to clean these items (as explained in Cleaning Procedure Guidelines p. 8). Use the results in the last column of the table above for the correct answers.

Finally, some photos show some poor practices or situations – ask participants to call these out:

Photo 2: cat allowed in ward
Photo 6: sink has grim and dirt around base of tap
Photo 8: cloths being dried over window frames
Photo 9: mops stored in bucket with head down

5. Involvement of parents

Parents of babies in the neonatal units play an important part in the process of care, be this providing breastmilk or practising Kangaroo Mother Care, for example. But such visitors to the unit also risk carrying germs which place the babies at risk of infections. The risk is increased because of their immature host defences and the frequent invasive procedures, which in turn increases the risk of mortality, morbidity, and prolonged hospital stay. Apart from parents' hands carrying germs, there are several other potential sources including clothing/textiles, personal jewellery, artificial &/or long nail, mobile phones, and outside footwear, as well as their respiratory hygiene which may necessitate mask-wearing and additional hand hygiene. Of course, parents who are unwell with contagious conditions, such as the common cold, should not come to the neonatal unit until they have recovered.

Parents may not know the risks they bring to their baby and it is crucial they are clearly informed of how best to reduce these risks with their behaviour. A poster or information sheets should be available to all visiting parents, and staff who observe poor practises – such as failure to wash hands or remove footwear, should take responsibility for informing parents of best practise and ensuring they adopt this. Environmental hygiene in the neonatal unit is everyone's responsibility.

Here is an example of the information given to parents with babies in the neonatal unit at a large hospital.

INFECTION PREVENTION IN THE NICU

NICU infants are at high risk for infection. You can help prevent the spread of germs that cause infections by ensuring that anyone who visits your baby follows these important infection prevention guidelines. Proper hand washing is a simple and proven way to reduce the risk of infection for all patients. All healthcare team members – doctors, nurses, parents, family and friends – are responsible for washing their hands before entering the NICU and upon entering your baby's room. If you are concerned that visitors have not washed his/her hands or used alcohol hand sanitizer on his/her hands, always feel free to ask.

Proper Hand Hygiene

- Before entering the NICU, wash your hands in the wash area located near the greeter desk.
- Remove all watches and jewelry. All jewelry can be a risk for infection so it is best to leave it at home.
- Fingernails should be no longer than 1/4 inch. Artificial nails and nail polish that is chipped should be removed. Germs on or under the fingernails can be harmful to your baby.
- Roll up your sleeves all the way to the elbows.
- Wash your hands and arms up to the elbows for one full minute. Sleeves should remain rolled up during your time in the NICU.
- Upon arriving at your baby's bedside, use the alcohol hand sanitizer to clean your hands again.
- Wash your hands with soap and water for 15 seconds or use alcohol hand sanitizer each time you re-enter the NICU.
- If you have twins or triplets, wash your hands with soap and water for 15 seconds or use alcohol hand sanitizer between handling each of your babies.

Wash your hands or use alcohol hand sanitizer

- For at least 15 seconds with soap and water after changing your baby's diaper.
- Before and after touching/holding your baby.
- Before and after using the breast pump in the NICU and at home.
- Before and after contact with food.
- After contact with any object, such as your cellphone.
- After using the restroom.
- After performing any personal hygiene.

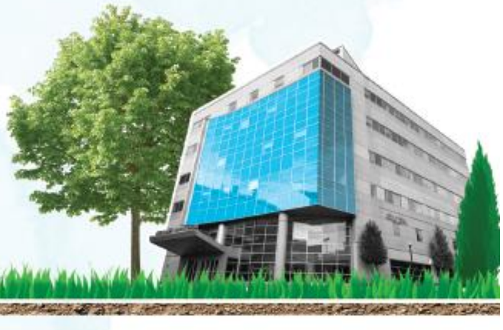

INFECTION PREVENTION IN THE NICU, CONT'D.

Illness

- All visitors must be healthy and have no signs of cold or flu symptoms (cough, fever, aches, runny nose), and no recent exposure to diseases that spread easily, such as chicken pox.
- If you have cold or flu symptoms, please do not visit the NICU until 24 hours after all symptoms have resolved. Please tell a staff member if you develop the flu or a cold while your baby is in the NICU.
- If you have a cold sore, a mask must be worn when you are with your baby.

Personal Items

- No plush toys or other toys should be placed in incubators or cribs. This not only poses an infection risk, it could also pose a potential risk for Sudden Infant Death Syndrome (SIDS).
- Photos from home may be placed inside the incubator after they have been secured in a plastic sleeve.
- Do not bring food into the NICU.
- Parents may bring water stored in a covered container to the bedside.
- Do not bring coats or tote bags into the NICU. Place these items in plastic bags located in the Family Wash Station across from the greeter desk.

If you have any questions or concerns about these guidelines, please speak with your baby's nurse or doctor.

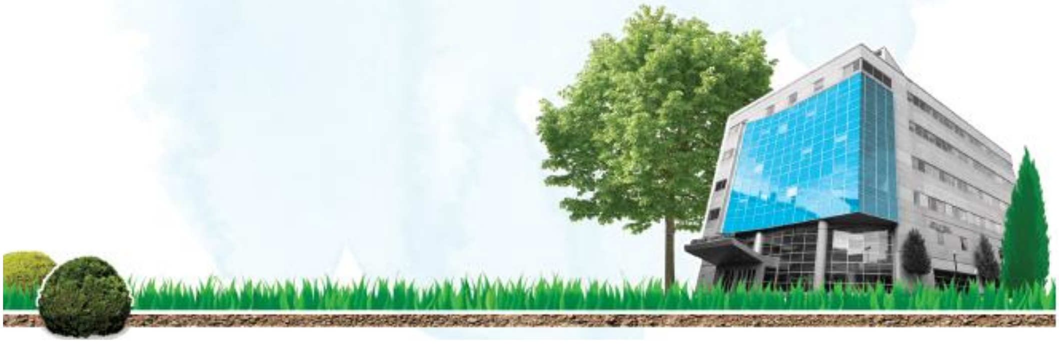

Discussion III

Encourage participants to discuss how this information could be adapted for their local neonatal unit. What advice is practical to apply? What should be changed? How could parents learn about these practises? Is a poster a good way to provide this information? How about parents who cannot read? Should this information be provided so parents can take it home to remind themselves?

? Cleaning the neonatal unit case study II

Adama had worked at the hospital for many years, mostly on the maternity ward. When she was asked to help out on the neonatal unit, she went straight away and started doing her usual practises that she had followed in the maternity ward. The nurses on the unit did not have time to instruct her, so an auxiliary was asked to guide Adama on what to do.

If you were that auxiliary, how would you have prepared Adama for cleaning?

What PPE should she wear? *(remind participants of what they learnt earlier in Clean Box 4)*

What should she be cleaning as part of the twice daily schedule?

How would you remind Adama of the patient zone?

How often should she be washing her hands?

Should Adama be encouraging visiting mothers to wash their hands before coming into the neonatal unit?

Use this final exercise to encourage participants to summarise what they have learnt across the entire module.

*****

Other resources

Infection Prevention and Control at Neonatal Intensive Care Units (healthynewbornnetwork.org)
https://www.healthynewbornnetwork.org/hnn-content/uploads/Infection-Prevention-and-Control-at-NICU-Slide-Deck-2.8.2018.pdf

https://newborn-health-standards.org/environmental_hygiene/

https://nijp.org/infection-control-protocol-in-nicu-suitable-for-a-peripheral-newborn-care-unit/

https://www.cdc.gov/hai/prevent/resource-limited/special-areas.html#anchor_1585593914670
