# Supplementary material for: Assessing the impact of a cleaning programme on environmental hygiene in labour and neonatal wards: an exploratory study in The Gambia
Source: Antimicrob Resist Infect Control. 2024 Apr 8;13:36. doi: 10.1186/s13756-024-01393-6 (PMC11003010; doi:10.1186/s13756-024-01393-6)
Supplement: Supplementary file 2 — Supplementary Material 2. [file 13756_2024_1393_MOESM2_ESM.docx]

| **Supplementary Table A1: Near-patient sites for dipslide collection** | | | | | |
| --- | --- | --- | --- | --- | --- |
| **Intervention Arm: Neonatal Unit** | | | **Control Arm: Maternity Unit** | | |
| **Location** | **S/No** | **Source/Object** | **Location** | **S/No** | **Source/Object** |
| Acute Care ward | 1 | Resuscitaire 1 (Right side) | Labour ward | 1 | Cubicle 1_bed (Right side) |
|  | 2 | Resuscitaire 1 (Left side) |  | 2 | Cubicle 1_bed (Left side) |
|  | 3 | Resuscitaire 2 (Right Side) |  | 3 | Cubicle 1_bed (Bottom) |
|  | 4 | Resuscitaire 2 (left side) |  | 4 | Cubicle 1_table/cabinet (Top surface) |
|  | 5 | Infusion pump monitor 1 (Front) |  | 5 | Cubicle 1_cot rail (Nearest side) |
|  | 6 | Infusion pump/monitor 2 (Front) |  | 6 | Cubicle 2_bed (Right side) |
|  | 7 | Cot 1 (Right side) |  | 7 | Cubicle 2_bed (Left side) |
|  | 8 | Cot 1 (Left side) |  | 8 | Cubicle 2_bed (Bottom) |
|  | 9 | Cot 2 (Right side) |  | 9 | Cubicle 2_table/cabinet (Top surface) |
|  | 10 | Cot 2 (Left side) |  | 10 | Cubicle 2_cot rail (Nearest side) |
|  | 11 | Cot 3 (Right side) |  | 11 | Cubicle 3_bed (Right side) |
|  | 12 | Cot 3 (Left side) |  | 12 | Cubicle 3_bed (Left side) |
|  | 13 | Cot 4 (Right side) |  | 13 | Cubicle 3_bed (Bottom) |
|  | 14 | Cot 4 (Left side) |  | 14 | Cubicle 3_table/cabinet (Top surface) |
|  | 15 | Cot 5 (Right side) |  | 15 | Cubicle 3_cot rail (Nearest side) |
|  | 16 | Cot 5 (Left side) |  | 16 | Cubicle 4_bed (Right side) |
|  | 17 | Cot 6 (Right side) |  | 17 | Cubicle 4_bed (Left side) |
|  | 18 | Cot 6 (Left side) |  | 18 | Cubicle 4_bed (Bottom) |
|  | 19 | Cot 7 (Right side) |  | 19 | Cubicle 4_table/cabinet (Top surface) |
|  | 20 | Cot 7 (Left side) |  | 20 | Cubicle 4_cot rail (Nearest side) |
|  | 21 | Cot 8 (Right side) |  | 21 | Cubicle 5_bed (Right side) |
|  | 22 | Cot 8 (Left side) |  | 22 | Cubicle 5_bed (Left side) |
|  | 23 | Cot 9 (Right side) |  | 23 | Cubicle 5_bed (Bottom) |
|  | 24 | Cot 9 (Left side) |  | 24 | Cubicle 5_table/cabinet (Top surface) |
|  | 25 | Cot 10 (Right side) |  | 25 | Cubicle 5_cot rail (Nearest side) |
|  | 26 | Cot 10 (Left side) |  | 26 | Cubicle 6_bed (Right side) |
|  | 27 | Incubator 1 (Front) |  | 27 | Cubicle 6_bed (Left side) |
|  | 28 | Incubator 2 (Front) |  | 28 | Cubicle 6_bed (Bottom) |
|  | 29 | Incubator 3 (Front) |  | 29 | Cubicle 6_table/cabinet (Top surface) |
|  | 30 | Incubator 4 (Front) |  | 30 | Cubicle 6_cot rail (Nearest side) |
|  | 31 | Ward round trolley (Top surface) |  | 31 | Triage room_bed (Right side) |
|  | 32 | Ward round trolley (Handle) |  | 32 | Triage room_bed (Left side) |
|  | 33 | Medication trolley (Top right side) |  | 33 | Triage room_bed (Bottom) |
|  | 34 | Medication trolley (Top left side) |  | 34 | Triage room_table/cabinet (Top surface) |
|  | 35 | Ward Table (Top surface) |  | 35 | Triage room_scanning machine (Handle) |
| Kangaroo Mother Care (KMC) Ward | 36 | Bed 1 (Right side rail) |  | 36 | Triage room_scanning machine (Keypad) |
|  | 37 | Bed 1 (Left side rail) |  | 37 | Nurse station_table (Top surface) |
|  | 38 | Bed 2 (Right side rail) |  | 38 | Nurse station_table (Drawer handle) |
|  | 39 | Bed 2 (Left side rail) |  | 39 | Materials trolley (Top surface) |
|  | 40 | Bed 3 (Right side rail) |  | 40 | Mobile CTG machine (Handle) |
|  | 41 | Bed 3 (Left side rail) |  |  |  |
|  | 42 | Bed 4 (Right side rail) |  |  |  |
|  | 43 | Bed 4 (Left side rail) |  |  |  |
|  | 44 | Ward Table (Top surface) |  |  |  |

**Supplementary Table A2**

**Cleaning Tool**

*Cleaning observation Cleaning Tool*

*Page 1 of 3*

Cleaning episode number

(Example 01)

**Cleaning quality observation tool**

Ward ID Labour ward


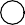

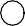


Neonatal ward

Zone Delivery Cubicle

Triage Room


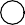

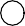


Zone Critical Ward

KMC Ward


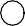

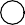


Cadre of person performing the cleaning Cleaner Nurse Student nurse Other

Don't know


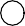

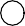

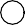

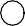

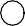


Cadre of person other (specify)

Date of Observation

(| | |/| || |/| || || || |(dd/mm/yyyy))

Start of the observation period: hh:mm

(hh:mm)

End of the observation period: hh:mm

(hh:mm)

Surface being cleaned (select one):

Select the type of surface being cleaned Bed Frame (Delivery cubicle) Mattress (Delivery cubicle) Bed side (Delivery cubicle) Bed Frame (Triage room) Mattress (Triage room)

Scanning machine (Triage room)


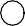

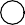

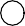

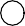

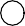

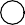

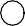

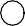

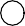

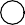


Bed side cabinet/locker (Triage room) Table (Nurse station)

Chair (Nurse station)

Other equipment (e.g. Materials trolley in labour ward; mobile CTG machine)

Select the type of surface being cleaned Resuscitaire (Critical Ward) Cot (Critical Ward) Incubator (Critical Ward)


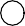

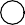

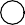

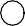

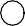

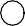

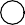

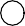

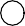

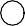

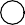


Infusion pump/Vital sign monitor (Critical Ward round/Notes Trolley (Critical Ward) Medication Trolley (Critical Ward)

Bed Frame (KMC ward) Bed Mattress (KMC ward) Table (KMC ward) Patient chair (KMC ward) Other equipment

Blood spill present (fresh or dried) (select one of No

the options below) Impossible to see


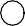

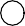

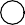


Yes

3a) For specific cleaning episodes related to the cleaning area (surface) selected above (high-level surface/beds)

1. PPE worn (gloves and apron) Yes

No


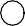

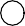

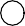


Impossible to see

1. Patient/staff present on the surface (can impede Yes

cleaning) No


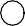

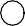

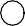


Impossible to see

1. Clutter/items removed prior to cleaning Yes No

NA (there is no clutter) Partial


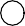

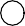

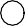

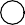

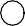


Impossible to see

1. Fresh cloth being used Yes

No


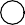

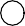

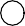


Impossible to see

1. Dampened the cloth in cleaning solution Yes No

Impossible to see


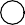

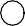

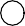


1. Folded the cloth to create several clean cloth Yes

surfaces/sides No


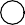

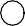

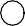

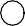


NA (very small surface) Impossible to see

1. Cleaned with the cloth using one swipe per cloth Yes

side No


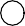

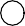

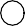


Impossible to see

viii .Replaced cloth if applicable Yes

No NA


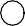

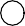

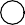

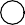


Impossible to see

ix.Double dipping Yes

No


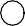

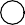

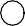


Impossible to see

1. Systematically worked from clean to dirty areas Yes No


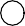

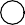

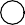

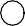


NA (dirty area not recognisable) Impossible to see

1. Systematically worked from high to low areas Yes No NA

Impossible to see


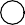

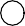

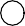

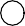


1. Cleaned all surface areas Yes

No


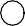

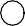

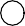


Impossible to see

xiii. Left area to air dry/dried Yes

No Partial


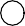

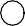

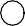

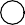


Impossible to see

Step 3b. For the specific cleaning episode related to the cleaning area selected above (if a blood spill is present)

1. PPE worn (gloves and apron) Yes

No


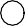

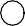

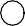


Impossible to see

1. Patient/staff present on the surface (can impede Yes

cleaning) No


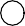

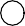

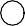


Impossible to see

1. Clutter/items removed prior to cleaning Yes No

NA [there is no clutter] Partial


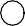

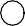

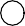

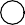

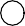


Impossible to see

1. Covered spillage with absorbent material Yes No Partial

Impossible to see


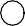

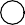

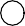

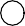


1. Removes absorbent material after absorption Yes No

Impossible to see


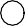

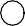

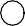


1. Uses fresh cloth Yes

No


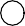


Impossible to see

1. Dampened the cloth in cleaning solution Yes No

Impossible to see

1. Cleaned with the cloth the spillage area Yes No

Impossible to see
